# Mobile instant messaging for rural community health workers: a case from Malawi

**DOI:** 10.1080/16549716.2017.1368236

**Published:** 2017-09-15

**Authors:** Christoph Pimmer, Susan Mhango, Alfred Mzumara, Francis Mbvundula

**Affiliations:** ^a^ Institute for Information Systems, School of Business, University of Applied Sciences and Arts Northwestern Switzerland, FHNW; ^b^ Millennium Promise, Zomba, Malawi

**Keywords:** Instant messaging, mHealth, WhatsApp, global health, community health worker

## Abstract

**Background**: Mobile instant messaging (MIM) tools, such as WhatsApp, have transformed global communication practice. In the field of global health, MIM is an increasingly used, but little understood, phenomenon.

**Objectives**: It remains unclear how MIM can be used by rural community health workers (CHWs) and their facilitators, and what are the associated benefits and constraints. To address this gap, WhatsApp groups were implemented and researched in a rural setting in Malawi.

**Methods**: The multi-site case study research triangulated interviews and focus groups of CHWs and facilitators with the thematic qualitative analysis of the actual conversations on WhatsApp. A survey with open questions and the quantitative analysis of WhatsApp conversations were used as supplementary triangulation sources.

**Results**: The use of MIM was differentiated according to instrumental (e.g. mobilising health resources) and participatory purposes (e.g. the enactment of emphatic ties). The identified benefits were centred on the enhanced ease and quality of communication of a geographically distributed health workforce, and the heightened connectedness of a professionally isolated health workforce. Alongside minor technical and connectivity issues, the main challenge for the CHWs was to negotiate divergent expectations regarding the social versus the instrumental use of the space.

**Conclusions**: Despite some challenges and constraints, the implementation of WhatsApp was received positively by the CHWs and it was found to be a useful tool to support distributed rural health work.

## Background

Mobile instant messaging (MIM) services have seen tremendous growth in the past few years. MIM is a relatively simple communication technology which offers real-time and asynchronous communication features. While instant messaging originated as text-based communication on stationary computers, technological advances have led to the enrichment of its multimodal capacities. Current features allow users to share audio, images and video across a range of mobile and non-mobile devices. In contrast to short message service (SMS), which incorporates point-to-point communication, instant messaging also enables group conversation, i.e. communication between multiple interactants in one digitally shared space. MIM platforms are also known as ‘over-the-top’ (OTT) applications, because they allow communication regardless of the network and the mobile device being used [1]. Key functions of instant messaging are alerting features such as sound or pop-ups that notify users of new messages, presence features that reveal information about the users’ current status, and additional profile information [2]. WhatsApp, which offers this functional repertoire to more than 1.2 billion active users per month, is one of the most popular MIM platforms [3].

From a communication perspective, the use of mobile and networked communication has an evolutionary meaning: it frees (bilateral) human communication from both physical proximity and spatial immobility [4], and allows groups and networks of highly distributed and mobile interactants to engage in more complex forms of communication, thus offering interesting features for a global and distributed health workforce [5]. The investigation of mobile networked communication technology represents, however, a relatively novel area in the study of global health, especially in low-income settings, which has so far focused on more restricted technologies, such as SMS [6–8].

To further substantiate the thin, empirical and conceptual understanding of the use and usefulness of MIM for global health, the implementation and use of WhatsApp groups by community health workers (CHWs) as a means to support healthcare work were investigated in this study, using the case of rural Malawi. CHWs play a central role in the setting under investigation and beyond. They provide basic health and health-promotion services regarding hygiene and sanitation, immunisations, growth monitoring, antenatal care, family planning and disease surveillance [9]. CHW programmes have been shown to be effective, and through ongoing educational and sensitisation activities they may even change the health beliefs of a community [10].

Although health professionals are increasingly using instant messaging services, only a limited number of studies has been carried out. Previous work has examined stationary [11] and, predominantly, MIM systems in the communication of clinical teams [12–14]. For example, Johnston et al. [12] documented how British emergency teams accessed WhatsApp groups to discuss clinical and administrative information. The participants found the use of MIM to be efficient and reported that it had flattened team hierarchies. Similarly, neurosurgical teams had high regard for WhatsApp, which they used as a tool to share patient-related information, including radiological images. This virtual information exchange was reported to decrease the time taken for morning handover and it helped remote team members to remain updated [14]. Khanna et al. [13] found that the use of WhatsApp by orthopaedic surgeons significantly increased patient-related awareness and also resulted in swifter handovers.

MIM has also been made available to health workers in disadvantaged or rural areas. For example, WhatsApp was provided to community health nurses in Ghana with the goal of increasing motivation and a sense of connection among professionals who work in isolation [15]. The conclusion, when WhatsApp was used educationally by undergraduate nursing students and educators in a primary healthcare module in South Africa, was that it supported nurses to integrate theory and practical experience [16]. Henry et al. [17] investigated how Kenyan CHWs and their supervisors started to use instant messaging groups to support supervision, professional development and team building. Their results indicated that the use of WhatsApp was highly regarded and taken up easily by the study participants. Most of the messages related to the supervision objectives of quality assurance and creating a supportive environment.

In essence, despite the increasing use of MIM, there is limited knowledge available on the ways in which MIM can be used by CHWs, and what are associated benefits and constraints. This is the gap that the present study seeks to address.

## Methods

### Research questions

The research design was operationalised in the form of an interpretive case study approach to understand the use of MIM tools through the meanings that the users (CHWs, senior CHWs and facilitators) assign to them [18,19]. Interpretive designs are well-established approaches for researching information and communication systems in healthcare [e.g. 20]. They pay particular attention to how users make sense of digital media in specific sociocultural and organisational contexts. Given the exploratory character of the study, the research questions were broadly defined as follows:

RQ1: How and for what purposes do health workers use WhatsApp groups in support of their work?

RQ2: What benefits and constraints do health workers associate with the use of WhatsApp groups?

### Research setting

The study was part of a larger, 8 month piece of field research on the use of mobile technology by CHWs, carried out within the Millennium Villages Project sites in rural Malawi. The Millennium Villages Project incorporated a community-led and integrated development approach which combined disciplines such as health, education, business, agriculture and infrastructure to tackle the root causes of rural poverty in resource-poor areas.

The research intervention was carried out in two rural sites which were centred around the two main clinics of the project. Whereas both clinics were linked to the national power grid, site A was more remote than site B, which was close to the main road. Health management processes and the organisation of the health workforce were identical, as both were managed under the auspices of the same project. The interventions reported in this paper lasted for about 5 months in total. However, the health workers and facilitators continued with the use of the WhatsApp group after the end of the research project.

### Study participants and intervention

This study involved CHWs [called health surveillance assistants (HSAs) in Malawi], senior CHWs, who were managing and supervising small groups of CHWs, and facilitators. Facilitators were more experienced health professionals, for example clinical officers, who supervised CHWs and senior CHWs and were responsible for specific subject areas, such as human immunodeficiency virus/acquired immune deficiency syndrome (HIV/AIDS) or nutrition. The Millennium Villages Project had equipped the study participants with basic smartphones 3 years before this study. CHWs were using the phone-based job-aid application CommCare as well as a toll-free line to communicate with the wider health team.

Starting with site A in April 2015, WhatsApp was installed on the CHWs’ smartphones. Site A’s most senior CHW created the WhatsApp group and invited fellow CHWs and facilitators to join. A short training session, lasting approximately 1 hour, was carried out for the participants. The topics included technical instructions, how to protect clients’ privacy (e.g. not sharing patient-identifying information), advice on how to use WhatsApp selectively to save the battery, and a warning not to share any offensive and/or discriminatory material. While socialising was permitted, the group was advised to focus on work-related use of the space. The same procedure was repeated in site B 2 months later. Owing to bandwidth restrictions, no voice calls were made via WhatsApp during the interventions.

The total number of participants in the two WhatsApp groups was 41. The 21 participants in group A comprised one facilitator, four senior CHWs and 16 CHWs. The 20 participants in group B included two facilitators, two senior CHWs and 16 CHWs. In site A, there were 16 male and five female participants, whereas in site B the number of female participants exceeded the number of males, with 14 and six, respectively. According to the preliminary survey, the average age of CHWs was similar in both groups, with a mean of 30.94 years in site A (*n* = 18, SD = 6.83) and 32.44 years in site B (*n* = 16, SD = 3.52). The CHWs’ perceived competencies in their ability to use the mobile phone was 4.30 on a Likert scale ranging from 1 (strongly disagree) to 5 (strongly agree) (*n* = 20, SD = 1.22) in site A and 4.21 (*n* = 19, SD = 1.03) in site B.

### Data gathering and analysis

The two main data sources were (1) written conversations in the WhatsApp groups and (2) focus groups with the CHWs and interviews with facilitators. Although each of the two sources was used for both research questions, the WhatsApp content served as the central reservoir to respond to RQ1, whereas the interviews and the focus groups constituted the main source for RQ2. In addition, RQ1 was supported by quantitative analysis of the WhatsApp communication patterns and RQ2 was supplemented by two open survey questions asking CHWs about their perceptions of WhatsApp. The four data resources and the respective methods of analysis are presented in detail as follows.

First, thematic content analysis of the WhatsApp conversations was conducted to understand the nature of information exchange, i.e. the forms and purposes of its use (RQ1). As the WhatsApp discussions were in both English and Chichewa (the local language), the anonymised conversations in Chichewa that took place in each group in the first 2 months were translated into English. This allowed the non-Chichewa-speaking researcher to analyse the data.

In addition, and as a means to examine basic quantitative communication patterns, the content of the WhatsApp groups of approximately 5 months of conversations was extracted and analysed using SPSS version 22 for the statistical analysis (site A: 15 April to 14 June; site B: 10 June to 4 September). In total, 2059 messages (*n* = 845 in site A, *n* = 1214 in site B) were collected and quantitatively analysed. Measurements included the average number of monthly messages per group and participant, and potential differences in terms of written contributions relating to group or gender.

Secondly, to elicit the participants’ perceptions of the benefits and constraints of WhatsApp (RQ2), individual interviews were carried out with each of the three facilitators (one facilitator participated in both groups), and four focus groups with 21 CHWs (43 min duration, on average) were conducted in June and July 2015. The interview and focus group guides included a broad set of questions referring to the forms, patterns and motivations of participation, and to the perceived benefits and constraints. The questions for CHWs and facilitators were the same. For example, the question ‘Was the participation in the WhatsApp group useful regarding your work? Why or why not?’ was discussed with CHWs in the focus groups and with facilitators in the individual interviews. In addition, the facilitators were asked about forms, patterns and motivations that they observed in the CHWs they supervised. For example, regarding the perceived benefits, facilitators were asked about whether or not they observed that the CHWs found participation in the WhatsApp groups useful for their work.

The approach was semi-structured. That is, the question guide was followed with flexibility, allowing for new themes that emerged during the conversation to be discussed [21]. With the participants’ agreement, all conversations were audio-recorded, notes were taken during the conversations and the audio episodes were transcribed verbatim by a professional translation service. In support of RQ2, a short survey was carried out. The survey consisted of two open-ended questions asking CHWs about (1) what they liked and (2) what they disliked the most about using WhatsApp. The paper-based survey was administered during the weekly coordination meetings and involved nearly all CHWs who participated in the interventions (site A, *n* = 19 out of 20; site B, *n* = 17 out of 18).

The interview transcripts, the answers to the survey questions, and the written and translated conversations of the two WhatsApp groups were entered into the software NVivo8. Considering the limited level of former knowledge, an inductive approach was used [22] following principles of an interpretive design.

In the open coding phase, the complete data set was read and the material was labelled according to the themes that emerged within the main domains specified by the research questions (purposes of use, benefits and constraints). The subcategories were descriptive and close to the empirical data. Different subcategories were further grouped and the relationship between these categories was examined [22]. The first author read and analysed the whole data set. Each of the three coauthors analysed approximately 30% of the data. The emerging categories were developed independently, and were repeatedly discussed and refined among the research team until consensus was reached. Finally, the findings were contrasted with related literature.

## Results

### Purposes and forms of use

The qualitative thematic analysis of the WhatsApp group conversations and of the interviews and focus groups revealed two main purposes of use, instrumental and participatory, with three subcategories each.

#### Instrumental purposes

The first main category, instrumental purposes, encompasses the deliberate use of the digital space to achieve health-service and project-related goals.

*Mobilising resources*: The content analysis showed that facilitators and senior CHWs used the group space for vertical task division and resource allocation, mapping tasks to individual CHWs or the whole group in a top–down format. Examples include mobilising human resources for meetings, training and health-promotion campaigns, and allocating material resources, such as drugs or solar chargers. The quote below illustrates written instructions in the WhatsApp group provided by a facilitator regarding the preparation for a campaign and acknowledging responses from CHWs.

[the campaign] will include measuring length in under 2 children alone. Special forms for recording the length of the children will be provided. All HSAs [CHWs] will then be required to enter […]. … Please arrange for height boards or lengthmats among yourselves

10:59, May 9 – CHW7: Thnx bossv

14:42, May 9 – CHW11: Thanks, we will do bebeautiful job […]

(WhatsApp conversation)

In addition, the content analysis revealed that CHWs used the space for lateral task division and also requested information from senior CHWs and facilitators. This made the information exchange multilateral and cross-hierarchical, involving both pull and push mechanisms.

*Problem solving and information sharing*: The participants used WhatsApp to identify and discuss health-related as well as technical problems, and to develop and share attendant solutions. A simple example of this included a CHW who ran short of airtime and was helped out by his colleagues. Another example included redistributing polio vaccines after identifying a lack of vaccine in one area. In addition to problems brought in from ‘outside’, the group conversations proved to be a primary source for identifying work-related challenges. For example, in a conversation where a facilitator encouraged CHWs to use a stopwatch app to measure fast breathing in children, it was found that, in contrast to what had been assumed, no such app was available. These apps were subsequently installed. Facilitators and senior CHWs also responded to day-to-day health questions from CHWs, for example, regarding food groups. To a lesser extent, CHWs shared lessons learnt and insights from their daily work with the group, with an example being sharing good practice for treating malnourished children. In the interviews, facilitators conceived the opportunity to share identified solutions with the whole group as one of the group’s key features:

Yes for me, yes I’ve had some areas, maybe one HSA [CHW] can experience a problem […] and at the same time I can share with them all [all CHWs] at once so that they can also learn from what this one HSA experienced. [Interview, facilitator]

*Visualising and acknowledging performance*: Facilitators started using the digital space to share data regarding the CHWs’ performance. This monitoring information was extracted from the mHealth app CommCare, which CHWs use to collect data during household visits. For example, a facilitator shared individual and total performance statistics for monthly household visits and monthly salaries, including bonuses, in both of the groups. Originally, all data and experiences were communicated during the weekly meetings, and this process shifted from face-to-face settings to the social media space. Facilitators and CHWs used the performance data as a source for mutual acknowledgement and motivation, as the next extracts show:

Facilitator: 85% [household coverage] is alot! Congratulations Keep it up guys! Just don’t forget [to measure and enter the] MUAC [mid-upper arm circumference as a measurement to detect nutrition status]

CHW3: We Will continue working hard&hard, … we are proud of you [directed to facilitator]

(WhatsApp conversation)

#### Participatory purposes

The second main category is ‘participatory’ purposes, which, in contrast to managerial and task-related activities, revolve around CHWs’ participation in a socio-professional community. These include enacting (em)phatic ties, disclosing and accumulating claims, and engaging in cross-boundary debates, which emanate through the health workers’ social and political participation in the media space.

*Enacting (em)phatic ties*: As the analysis of the WhatsApp groups revealed, a central category was made up of communicative episodes in which CHWs, senior CHWs and facilitators reconnected socially and emotionally, for example, by greeting each other in the morning or saying goodnight, asking about each other’s work or conveying birthday wishes. The most overt form of empathy was emotional and spiritual support when CHWs fell sick or were injured. The underlying goal of many of these communications was not information exchange but rather mutual reassurance of the (virtual) co-presence of the group distributed in geographical space, as the next statement illustrates:

18:54, Jun 6 – CHW1: People, where are you?

18:55, Jun 6 – Senior CHW: No, we are here Ma’am

18:56, Jun 6 – CHW1: Ok I see [!] you

(WhatsApp conversation)

*Disclosing and accumulating demands*: CHWs also started to use the group for micropolitical purposes about work-related claims. By disclosing and repeating challenges and demands related to the accomplishment of their work, such as the lack of solar chargers, they could make their voice heard more easily. An example from health service delivery showed how CHWs pointed to financial resources needed for their work. This reminded and helped to alert senior CHWs and facilitators, who were also in the group. These needs were expressed directly (‘CHW7: Supervisor, where is the money issue of praz [allowance for drug distribution] on now?’) and indirectly. In particular, humour was often used as a moderator for uttering needs. This pattern came to the fore not only through the WhatsApp content analysis, but also in the focus groups: CHWs explicitly acknowledged their tactic of voicing their needs in a soft and humorous, but consistent way towards senior CHWs and facilitators, who also confirmed these practices:

… so they started discussing it [financial resources needed] … it was their way of communicating to us as their supervisors to say we should push, it was for a week, talking …. [Interview, facilitator]

*Engaging in cross-boundary debates*: The WhatsApp group also harboured discussions that went beyond health service delivery and health promotion, including themes such as culture, religion, cuisine, sport and politics. This rendered the space a broader news and discussion portal. Examples included sharing news from their district along with news about lifestyle, well-being and religious practices. Political dynamics were also debated, for example, the xenophobic incidents in South Africa and the political situation in Zimbabwe. Health-related aspects were interwoven into many of these broader debates as, for example, the next excerpt from a sports discussion illustrates. The CHWs interlaced central themes from their daily health practice [such as distributing oral rehydration salts (ORS) or painkillers] into an ongoing debate about an upcoming sports event.

CHW13: Before you start watching the Flames [The Malawi national football team] make sure you do the following …

1. Make sure your BP [blood pressure] is tested to avoid sudden death

2. Get some painkillers to prevent severe headache

3. Buy ORS to prevent diarrhea

(WhatsApp conversation)

#### Quantitative communication patterns

According to the quantitative content analysis of the WhatsApp groups, communication patterns were similar in both groups, with an average of 13.85 messages shared per day in site A and 13.95 messages in site B. Calculating the messages that were shared on average per participant per month, again similar patterns were found between the groups, with 20.06 (SD = 22.94) in site A, and 21.22 (SD = 23.33) in site B. A Mann–Whitney test confirmed that the monthly number of contributions per participant was not significantly higher for site A (median = 13.96) than for site B (median = 12.94) (*U* = 190.0, *p* = 0.602, two tailed). Within both groups, the range of monthly contributions varied considerably, from 1.00 to 96.24 messages in site A and from 2.80 to 103.49 messages in site B. Regarding the impact of gender (*n* = 22 male CHWs, *n* = 19 female CHWs) on the number of monthly contributions, no significant differences were found between male (median = 13.45) and female CHWs (median = 12.94) using a Mann–Whitney test (*U* = 192.5, *p* = 0.666, two tailed).

### Benefits and constraints

#### Benefits: manageability and connectedness

As the analysis of the interview and focus group data and the open-ended survey questions revealed, the positive perception of MIM was, in part, grounded in its usability aspects. This was particularly recognised through a number of statements in the survey such as: ‘it’s easy to use’ or ‘it’s user friendly’. More centrally, the value of MIM was associated with its ability to integrate the coordination of distributed resources in one communication space, most prominently by allowing the whole team to be reached at once in a reasonable time-frame. This quality can be compared to the previously time-consuming one-to-one communication chain when information was passed in the form of phone calls from the facilitator to the most senior CHW of the respective clinic, then to the other senior CHWs, who finally forwarded it to the individual CHWs. These processes were particularly cumbersome in view of the repeated efforts needed to reach CHWs who were temporarily unavailable because of a lack of power or network coverage. The previous ways of communication were also conceived to be more error prone, resembling the dynamics of ‘Chinese whispers’, as a facilitator indicated in the interview: ‘The message might change [in the process of forwarding it], yes. That happens a lot …’. MIM communication was no longer dependent on intermediaries and potential misconceptions were clarified earlier. The simple act of posting a message was valued because the responsibility for forwarding the information was delegated to the tool, making repeated calls obsolete. In essence, MIM helped to make a geographically distributed workforce more manageable. A facilitator explained this in respect to organising a campaign:

When I give a message I feel it’s much better than giving the message to the senior. When I put it on WhatsApp I know everybody is reached […] For the child happy days they [CHWs] were supposed to take the length of under two years children. I just gave them the message on WhatsApp and everybody was able to do that. [Interview, facilitator]

From the perspective of those receiving the information, enhanced communication was tied to MIM’s capabilities to integrate online and offline discussions, for example, allowing CHWs to access and contribute to past conversations. Supporting this perception, CHWs valued the heightened levels of information circulation among peers, senior CHWs and facilitators, which they linked to an increased awareness of work-related activities of the other CHWs beyond their own clusters:

We know what is […] going on in every cluster using WhatsApp. [Focus group, CHW]

We are getting updated to anything that is happening out there since we are using the WhatsApp. [Focus group, CHW]

The perception of the WhatsApp groups as information and knowledge platforms was also manifested in statements such as ‘We also learn a lot from WhatsApp’. The focus group analysis revealed that the ongoing interactions of information among participants cumulated in a more steady feeling of connectedness. The WhatsApp group extended the communicative terrain of the CHWs who tended to work in relative professional isolation, meeting only once a week as a group. As could be seen also in the analysis of the WhatsApp content, the platform allowed them to ‘gather’ in between these regular meetings with lengthy, parallel and discontinuous streams of discussion which extended past typical working hours into the CHWs’ personal spheres. This was deemed to nurture a feeling of virtual togetherness, as expressed by a CHW: ‬‬‬‬‬‬‬‬‬‬‬‬

it’s like since we just stay in the different areas and maybe it takes us a week to meet […], so when you see your friend posting […], oh it’s like you, it’s like getting us together …. [Focus group, CHW]

#### Constraints

Although media literacy was reported to be a minor challenge in the focus groups, the small number of written contributions by some of the CHWs was explained in the interviews by their lack of technological competency (i.e. typing and sending messages). A more prevailing constraint for the whole group was finding the right balance between instrumental, work-related and more social conversations. Reconciling the different expectations among participants was an ongoing negotiation process. While forms of use were, for example, discussed during the weekly meetings, tensions remained until the end. Twelve CHWs stated in the final survey that receiving inappropriate, non-work-related messages was what they disliked the most about MIM. However, completely banning social communication was deemed to be counterproductive by the facilitators, because of the risk of diminishing the acceptance and use of this inherently social, peer-to-peer communication medium. Instead, facilitators acknowledged that WhatsApp’s success as an instrument for global health was grounded in an ongoing calibration process, which required their guidance:

… prohibiting them [CHWs] completely on the social aspect it might even end up like frustrating them and then they might not even use the media […] We [the facilitators] need just to put in place the measures on how best they can balance that. They should socialise but at the same time they should really make use of this media for work related issues. [Interview, facilitator]

Some minor concerns were raised by CHWs who criticised the use of MIM during late hours. Late postings were deemed especially inappropriate in controversial debates, as those who had switched off their phones could not respond to allegations in a timely manner. This tension can be connected with the micropolitical pressure that some senior CHWs and facilitators found themselves exposed to when CHWs used MIM to express work-related needs, as one facilitator exemplifies:

they felt like that was the forum, they could express what is burning inside, yes, so I didn’t blame them, but I thought it was not good. [Interview, facilitator]

Another challenge was that although the CHWs read the facilitators’ organising and instructional messages, the majority tended not to acknowledge receipt of them. CHWs also criticised the lack of immediate comments and responses by some of their peers, which was linked to a poor power supply. Issues with the power also resulted in some CHWs being able to use WhatsApp only sporadically. Given the linear way in which WhatsApp constructs reading paths, delayed responses also manifested in parallel discussions. That is, contributions were sequentially strung together, one after another, without the ability to build thematic structures (e.g. in the form of threads). Although simplicity is a key feature of the tool’s success, it also inhibited the continuity and depth of discussion, as one facilitator remarked regarding the sharing and discussion of nutrition guidelines.

I think they forget [the guidelines], because sometimes there were conversations changed on the WhatsApp group. You could see we’ve already diverted from this [debate on nutritional guidelines] instead of concentrating, yes. [Interview, facilitator]

## Discussions

**Figure 1.**
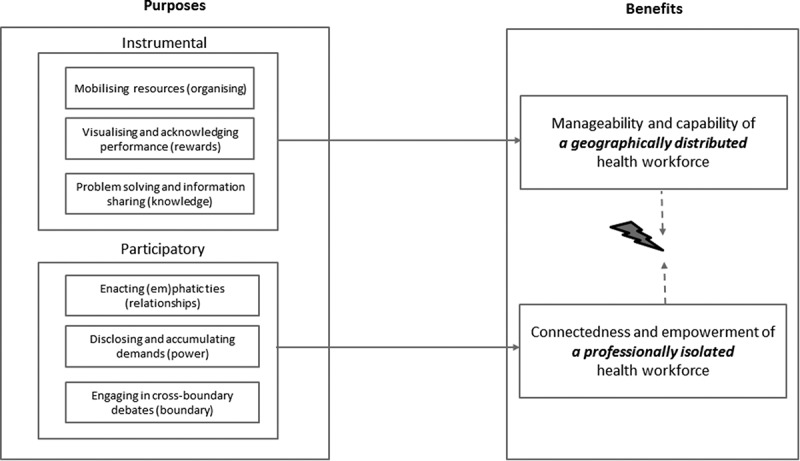
Purposes and benefits of mobile instant messaging for geographically distributed and professionally isolated health workers.

In the following sections, the results of this study are linked to previous communication concepts and literature on global health. It is worth reiterating that the use of the instant messaging tool was received well by CHWs and their facilitators and was used regardless of gender. The purposes of MIM use and attendant effects are synthesised in Figure 1 and discussed in the following sections.

### Instrumental communication for a distributed health workforce

Mobilising resources (organisation), visualising and acknowledging performance (rewards), and problem solving and information sharing (capacity) are key to the functioning of any organisational unit [23]. Integrating the coordination of scattered resources, people and materials in one extended communication zone can be linked to an enhanced manageability of the target group. This is of particular value in settings where the management of health workers is inhibited by geographical distances and lack of transport, a phenomenon which is typical for many global health contexts [24,25]. In addition, the use of MIM to visualise and acknowledge performance (beyond exclusive control and monitoring purposes), which has been revealed in this study, is relevant in view of the widely acknowledged lack of supervision tools and practices that facilitate recognition and feedback mechanisms in global health work [24,26,27].

The perceptions of MIM as a capability enabler were grounded in the facilitators’ information sharing of health and management guidelines and in its use for problem solving. The second aspect helped facilitators with continuous visualisation and problem solving beyond the geographical and temporal constraints of on-site visits, which are also common beyond the setting of this study [28]. The findings of this research indicate that through the use of MIM, supervisors and facilitators could trace developments in their groups more continuously and develop a more persistent problem-based support. The sharing of knowledge in the group was, however, ephemeral, being intertwined with the vibrant stream of other discussions. Thus, it could never supersede the focused learning and teaching of on-site training or the concentrated attention of face-to-face supervision, but should be conceived as an additional layer. Nevertheless, the provision of an additional space for problem solving and knowledge sharing is certainly of value in the context under investigation, in a typical sub-Saharan African setting where health workers have limited access to knowledge resources [29], peers and supervisory support.

### Participatory communication for an isolated health workforce

‘Participatory’ purposes of enacting (em)phatic ties, disclosing and accumulating claims, and engaging in cross-boundary debates came about through the health workers’ socioprofessional participation in the media space. These impacted on the feelings of connectedness of a professionally isolated health workforce. The lengthy, parallel and discontinuous streams of discussion that reached into the participants’ private time zones can be associated with new forms of professional connectedness. The patterns observed resemble what Timmis [30], drawing on Habuchi [31], described in her instant messaging study of students as ‘telecocooning’: the creation of a (new) zone of intimacy in which people can continuously maintain their (professional) relationships. Intimacy was not exclusively a product of the participants’ interactions. In addition, it was created through ‘outeraction’, borrowing a concept from Nardi et al. [32]; that is, the establishment of a sense of social proximity through ostensibly purposeless communication, for instance the reassurance of virtual co-presence. Although unrelated to primary health goals, these communication practices are highly relevant for rural and mobile health personnel whose work realities are typically disconnected from those of fellow workers, supervisors and facilitators, and who are dramatically affected by professional isolation [33].

The use of social media as a political instrument that facilitates horizontal connectivity in social mobilisation is not limited to this study, but has been reported in previous work, most prominently, perhaps, with respect to the so-called ‘Arab Spring’ [34]. With CHWs who uttered needs related to the accomplishment of their work, the dynamics observed in the digital space were not only lateral, but reached upwards through the hierarchy. A similar, though even more intense, substantiation of political power was reported from Taiwan, where more than 1000 emergency health personnel started articulating their professional concerns (about overcrowding) in a Facebook group. The protests soared and even the health minister participated in the group, breaking down previously impermeable hierarchical structures [35].

### Quality and limitations of research

Trustworthiness in this study was achieved through triangulating data, methods, investigators and theory [36,37]. The triangulation of data sources resulted from the involvement of two intervention sites and health workers in different roles (CHWs, senior CHWs and facilitators). Methodological triangulation was grounded in the blend of observational and perceptional data, such as the analysis of the written interactions and of interviews/focus groups and surveys.

The use of well-established research methods added to the study’s credibility [38]. Investigator triangulation was achieved by involving investigators from different backgrounds, such as from social sciences and public health, who analysed the data independently. In addition, and similarly to Pimmer et al. [39], the cooperation between ‘insiders’ and ‘outsiders’ was found to be highly valuable. ‘Insiders’ (i.e. local researchers from the project) were able to interpret the findings in light of existing sociocultural practices, while ‘outsiders’ questioned aspects that ‘insiders’ would have taken for granted. This further contributed to the study’s credibility because it ensured familiarity with the culture of the participating organisations [38]. Theoretical triangulation resulted from comparing the emerging concepts with frameworks and data from extant literature. To conclude, each of the research questions has been addressed by involving one main and two supplementary sources and thus the findings can be deemed to be relatively robust.

However, limitations in the study design are linked to the duration of the intervention, the specific context in which the study was carried out and the nature of outcomes reported. Future research which examines the long-term use of MIM, and which studies the practices and effects of MIM use in further cultural and organisational circumstances is thus required. In addition, quantitative measures are needed to further measure and corroborate some of the qualitative indicators identified in this analysis, such as professional connectedness.

### Practical considerations

The forms and purposes of use identified can help health workers and project managers in setting up similar designs. The flexible and dynamic forms of instrumental and participatory communication observed in this study can be seen as valuable ‘add-ons’ to more structured and predefined mobile health data collecting systems. A quality of the use of WhatsApp in the field of global health can be seen in its capacity to help geographically distant supervisors, programme managers or district health officers to obtain a better understanding of the CHWs’ day-to-day trials and tribulations. In this sense, social media spaces can be used as a qualitative seismometer, tracing the motions and emotions of remote and distributed rural health workforces. Given the broad and rapidly increasing availability of MIM, the WhatsApp group design (as investigated in this study) can be scaled up in many other global health settings. To leverage the potentialities of MIM for global health work and in primary healthcare settings more systematically, programme managers and policy-makers need to be brought on board as ‘active’ instead of ‘passive’ partners.

However, although the implementation of MIM in rural health systems in sub-Saharan Africa can be a worthwhile endeavour, it is certainly no panacea for the coordination of health and health-promotion work. Although the MIM tool in use was able to integrate online and offline conversations, issues regarding power and connectivity remain. In addition, the use of group messaging tools requires individual media skills. Although for most of the study participants the use of the technological spaces presented no problem, ‘mobile literacy’ was a challenge for a few. To ensure equitable access for all health workers, specific support needs to be offered in a sensible manner without exposing less technologically skilled health workers in front of the group. One approach from this study came through facilitators who observed participation patterns over time and offered individual and discreet support to health workers with restricted levels of contribution.

In addition to individual skills, the inherent tension between the realisation of instrumental and participatory purposes needed to be constantly negotiated by all group participants. The use of MIM spaces was found to be an ongoing learning and calibration process for the whole group. To leverage fully the space’s potential for global health communication, the participants needed to address instrumental and participatory ends simultaneously. A space used exclusively for instrumental purposes not only would minimise its potential for ameliorating professional isolation (and inhibiting potential power dynamics), but may even collapse as health workers would lose interest and abandon the space. To this end, it turned out to be valuable to agree on a number of ground rules regarding the content and style of contributions, explicitly allowing forms of socialising. It could be observed that the guidelines that were presented and discussed in the introductory training were followed by the CHWs. The WhatsApp analysis revealed no breaches of clients’ privacy, and no discriminatory material was shared. However, the facilitators deemed it helpful to be able to address and discuss tensions, which revolved around balancing work and social topics and the timing of the messages, in the weekly meetings. On very rare occasions, they also used these meetings to point to other inappropriate content, such as in one case in which witchcraft beliefs were stated.

It is also important to keep in mind that digital technology tends to reproduce rather than transform existing structures. In this sense, MIM would not compensate for the effects of bad supervision. Rather, it would worsen such effects, because in addition to technical and social skills, supervisors and CHWs would also need to develop media literacy skills to handle the dynamics of supervision in a digital space.

## Conclusion

WhatsApp groups which were introduced in a rural and low-resource community health setting were used by health workers for instrumental (e.g. mobilising health resources) and participatory (e.g. the enactment of emphatic ties) purposes. The identified benefits were centred on the enhanced ease and quality of communication of a geographically distributed health workforce, and the heightened connectedness of a professionally isolated health workforce. Despite some challenges and constraints, the implementation of MIM was received positively by the CHWs and was found to be a useful tool to support distributed rural health work.

## Paper context

Despite the popularity of mHealth and the increasing use of WhatsApp, very little is known about the use and usefulness of these platforms in rural health settings. This study found that community health workers in rural Malawi used WhatsApp groups for instrumental and participatory purposes. The benefits were centred on the enhanced ease and quality of communication and on heightened professional connectedness. Challenges included minor technical issues and balancing instrumental and social use.
